# Self-Organized Fast Routing Protocol for Radial Underwater Networks

**DOI:** 10.3390/s18124178

**Published:** 2018-11-28

**Authors:** Waheeduddin Hyder, Javier Poncela, Miguel-Angel Luque, Pablo Otero

**Affiliations:** Department of Ingeniería de Comunicaciones, University of Malaga, 29010 Malaga, Spain; waheed@uma.es (W.H.); luquen@uma.es (M.-A.L.); pablo.otero@uma.es (P.O.)

**Keywords:** underwater, UWSN, location free, routing protocol, self-organized, self-configured, wireless networks, proactive

## Abstract

An underwater wireless sensor networks (UWSNs) is an emerging technology for environmental monitoring and surveillance. One of the side effects of the low propagation speed of acoustic waves is that routing protocols of terrestrial wireless networks are not applicable. To address this problem, routing strategies focused on different aspects have been proposed: location free, location based, opportunistic, cluster based, energy efficient, etc. These mechanisms usually require measuring additional parameters, such as the angle of arrival of the signal or the depth of the node, which makes them less efficient in terms of energy conservation. In this paper, we propose a cross-layer proactive routing initialization mechanism that does not require additional measurements and, at the same time, is energy efficient. The algorithm is designed to recreate a radial topology with a gateway node, such that packets always use the shortest possible path from source to sink, thus minimizing consumed energy. Collisions are avoided as much as possible during the path initialization. The algorithm is suitable for 2D or 3D areas, and automatically adapts to a varying number of nodes, allowing one to expand or decrease the networked volume easily.

## 1. Introduction

An underwater wireless sensor networks (UWSNs) is an emerging technology for environmental monitoring and surveillance. Electromagnetic waves are not suitable for this transmission medium because of their high absorption rate. Acoustic waves provide better performance in this regard. However, the propagation speed of acoustic waves is only slightly larger than 1500 m/s, varying with temperature, depth and salinity, which significantly increases the propagation delay. Routing protocols designed for terrestrial wireless sensor networks (WSNs) are difficult to be adopted for UWSNs due to this physical constraint, as they assume virtually instantaneous propagation, thus allowing for more signaling overhead. The propagation delay is a constraint that must be handled at the medium access control layer, which will try to utilize the channel as much as possible. Additionally, acoustic transmission data rates are much lower than in aerial transmissions.

Terrestrial protocols for ad-hoc networks often rely on location information of the nodes, which, for UWSNs, is at best a challenging task, due to the unavailability of underwater positioning systems. Routing protocols for underwater sensor networks, which are based in the location information of the nodes, usually require the existence of a special node, a sink node that is equipped with a global positioning system (GPS) receiver to ascertain its location. The location of the rest of the nodes is estimated with reference to the sink node. Range-based schemes use time of arrival (ToA) [1,2] or angle of arrival (AoA) [3] techniques to estimate the distance between the nodes and, eventually, the location of the nodes. These techniques require additional devices to measure the distance, which makes them bulkier and more energy demanding. They also require time synchronization, which is another challenging task for underwater networks [4,5]. Autonomous underwater vehicles (AUVs) based protocols [3,4,5,6,7] typically use an AUV to visit the sensor nodes and collect the data to be transmitted to the gateway. The disadvantage of this scheme is obvious, as it requires additional resources to operate the AUV.

**Figure 1:**
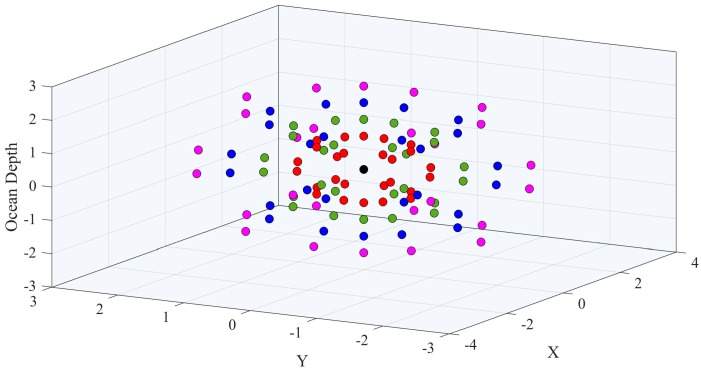
Example of node positions in a 3D spherical Wireless Sensor Network.

A radial topology can be deployed in 2D or 3D, as shown in Figure 1, and can be used to monitor sea volumes such as delta plumes, navigable straits, and archaeological sites as well as to secure facilities such as harbors, trade routes, mining sites, etc. It can also be of high value to monitor the surroundings of geological activity, such as the underwater volcano Tagoro, which erupted recently near island El Hierro [8,9]. The possibility of real-time video transmissions over these topologies have been analyzed in Reference [10]. In terms of interference, the worst case is when adjacent strings are parallel to each other, and this will be the case considered.

Routing is not an easy to solve problem in networks similar to the example of Figure 1. In this paper, we describe a location free routing protocol for 2D/3D underwater radial networks. The algorithm is designed to recreate the physical topology in the logical routing tables. The paths are established in such a way that only the nodes located in the same radius (called strings, see Section 3) will forward the data packets coming from other nodes which are farther from the sink. This way, all the packets are transmitted via the shortest paths. The algorithm uses the knowledge of the topology to initialize the paths. It is designed to avoid collisions and minimizes the overhead due to signaling. All together, these characteristics help save as much energy as possible.

This structure also helps to balance traffic among nodes that are at the same distance from the gateway such that energy depletion is as homogeneous as possible. Other proposals do not consider the physical topology when building the routing trees, and thus they do not balance traffic among routes, causing faster energy depletion of some nodes than others. Obviously, nodes closer to the gateway will forward a larger number of packets, using up energy faster than nodes located farther, which cannot be avoided

Our proposal uses a cross-layer approach by merging some steps of the route creation process with the medium access control layer, in order to minimize the odds of packet collision. The algorithm self-adapts to different network sizes and number of nodes, and, for example, would allow one ship to drop the nodes into the sea and they would find their neighbors and automatically create the paths. It minimizes the cost, in time and computational effort, of forwarding a packet and requires minimal periodic maintenance of the routes.

The rest of the paper is organized as follows. Section 2 discusses existing routing protocols. The overview of our proposed protocol is described in Section 3. Details of the protocol are given in Section 4. In Section 5, the packet formats are described. Section 6 mathematically analyzes the performance of our proposal. Section 7 contains the simulation results for the validation of the protocol’s algorithm.

## 2. Related Work

Routing protocols can be classified in two broad categories, namely, proactive and reactive. In proactive protocols, the forwarding nodes already know the next node to which the packet has to be forwarded, which reduces the forwarding time. Proactive protocols are, thus, more suitable for delay sensitive data. However, they have some disadvantages as well. They need to update the routing tables whenever the network topology changes or when node failures occur. Scalability is another problem of proactive protocols [11].

In reactive protocols, a node finds the next hop when it receives a packet to be forwarded [12,13,14,15]. This causes additional delay and makes this type of protocol unsuitable for delay sensitive data. However, periodic updates are not required, saving this overhead, and the network is scalable too.

Routing protocols can also be classified as location-based or location free. Location-based routing protocols choose the forwarding node on the basis of the location. For example, the next node can be chosen based on its distance to the destination or its depth. The additional measurement device increases energy consumption.

In the rest of this section, we present an overview of existing routing protocols. Although a taxonomic classification is hard to come by, we have classified the protocols, for the sake of presentation, in these groups: location free, location based, opportunistic, cluster based, energy efficient, mobility based and reliable data deliver protocols.

### 2.1. Location Free Protocols

The Network Layer Protocol for UANs (NLPU) [16] is a self-configured, proactive protocol based on the concept of full-duplex channel utilization. The gateway is the master node, manages the topology of the network and establishes the routes by means of a topology discovery probe. The probe assigns channel codes to each node it travels through along the network. Conflict remains possible if the nodes select the same channel. This issue is resolved by the common parent node. Once the probe completely tours the network, a completion notice is sent back to the gateway. The protocol heavily relies on full-duplex communication. 

The Location Free Link State Routing (LFLSR) [17] protocol addresses the problem of communication voids. Every node selects the next hop based on three metrics, namely hop count, path quality, and depth. A beacon message is broadcast from the sink node and forwarded along the network. This message is used to update the information of the paths. This protocol also requires a pressure measurement device, because the beacon message is forwarded to the neighbor closest to the surface for paths with similar quality.

### 2.2. Location Based Protocols

Vector Based Forwarding (VBF) is a location based protocol [18]. It introduced the concept of virtual pipes, a set of nodes from the source to the sink that can possibly forward the packets (a virtual circuit). Multiple nodes within the virtual pipe forward the data packets to counter packet losses and node failures. However, in case of networks with low density of nodes, voids may exist, which decreases the delivery ratio. The power consumption is mainly due to three-way handshaking. To solve the problem of voids, Hop-by-Hop VBF (HH-VBF) [19] proposes local virtual pipes, from one node to the next, rather than from source to destination. However, the signaling overhead is higher than in VBF. The Adaptive Hop-by-Hop Vector-Based Forwarding (AHH-VBF) protocol [20] is based on the HH-VBF protocol. AHH-VBF may change the direction of the virtual pipeline and its radius to restrict the forwarding range of the packets. As a consequence, the reliability of the packet delivery increases in sparse networks and duplicate packets are fewer than in dense networks. Power control is also employed. LB-AGR [21] routes the packets on the basis of the difference in available power, node density and proximity. Compared to VBF the packet delivery ratio is poorer, but the average energy consumption is significantly better. 

Directional Flooding-based Routing (DFR) [12] assumes that every node knows the locations of the next-hop node and the destination node. Forwarding nodes are selected on the basis of the link quality and angle of arrival. In Focus Beam Routing (FBR) [22], each node has location information of the final destination. The forwarding area is selected based on transmission power. If there is no node present in the forwarding area, the power is increased. Sector-Based Routing with Destination Location Prediction (SBR-DLP) [23] assumes that every node knows the movement of the destination nodes. Pre-planned movement makes implementation of SBR-DLP quite limited.

In the Location-Aware Routing Protocol (LARP) [24], the sink nodes find their location using GPS and broadcast their location coordinates. The other nodes calculate their location by using the reference of at least three sink nodes. When the location of a packet destination is not known, the sender queries the sink nodes. The sender finds the next hop by broadcasting the location of the destination node and a “moving direction” packet. If a receiving node finds that it is in the same direction, it replies, and the packet is forwarded. Hop-by-Hop Dynamic Addressing Based Routing (H2-DAB) [25] uses dynamic addresses for the nodes, such that they reflect their depth levels using shorter addresses for nodes closer to the surface.

### 2.3. Opportunistic Protocols

The Depth Based Routing protocol (DBR) [26] is based on opportunistic routing. Every node ranks the quality of the route and the next hop is based on the number of hops. There is no mechanism of recovery in case that there is no forwarding node in the direction of the sink node. If the depth of the neighbor receiving the packet is less than the depth of the sender, it will forward the packet. Nodes closer to the sink forward the packet first. However, communication void problems may occur if the nodes are very sparse. The Weighting Depth and Forwarding Area Division DBR (WDFAD-DBR) [27] protocol considers the depths of expected forwarding nodes to improve the delivery ratio, using a location prediction mechanism. The forwarding region adaptively changes according to the density of nodes and the channel conditions, to reduce the number of duplicate packets. Segmented Data Reliable HydroCast [13] is similar to DBR, but it addresses the problem of voids and local maxima. Each local maximum node maintains a recovery route towards a neighbor deeper than itself. It has a higher energy consumption compared to DBR. 

The Multi-Sink Opportunistic Routing Protocol (MSORP) [28] uses a two tier topology. The bottom tier includes the sensor nodes, the mesh nodes and the underwater (UW) sinks and the top tier contains the surface buoy and the monitoring center. Sensor nodes send their data to mesh nodes, which aggregate the data and forward it to the sink. Void-Aware Pressure Routing (VAPR) [29] uses depth information, hop count and sequence numbers to find the next hop and the directional path. Sink nodes start the localization process by transmitting beacon packets. In order to elude the voids in the network, the next hop selection is based on the data forwarding direction and the next-hop’s data forwarding direction. Efficient Opportunistic Routing Technology (EFFORT) [30] uses opportunistic end-to-end cost (OEC) to reduce the number of forwarding nodes, and thus increase the network lifetime. The OEC cost is determined on the basis of a forwarding node’s residual energy and link reliability.

Opportunistic Void Avoidance Routing (OVAR) [31] allows nodes which are farther away from the sink than the sender to take part in packet forwarding. However, the nodes closer to the sink node will have priority. This helps to elude the voids or local maxima problem.

### 2.4. Cluster Based Protocols

The Distributed Minimum-Cost Clustering Protocol (MCCP) [32] forms clusters based on the total energy required to send data to the cluster head, the residual energy of the nodes of a cluster, and the cluster heads’ relative distance to the sink. Each cluster head candidate constructs its set of neighbors. The costs of the clusters are exchanged to minimize the overall cost. MCCP uses a centralized approach, designating more cluster heads around the sink node to balance the traffic load and the energy consumption by the cluster heads. In the Distributed Underwater Clustering Scheme (DUCS) [14] protocol, the nodes keep rotating the role of cluster head among themselves to conserve energy. In the Temporary Cluster Based Routing (TCBR) [33], multiple sinks are used to increase the delivery ratio. Special nodes, called couriers, collect data and transmit it to one of the sink nodes. TCBR is not suitable for time critical applications.

Greedy Geographic Forwarding based on Geospatial Division (GGFGD) and Geographic Forwarding based on Geospatial Division (GFGD) [34] are 3D routing protocols. In GGFGD, first the target cluster is selected. The distance of the target cluster from the sink must be shorter than the distance from the current cluster. This may make the total path longer. Then, the next hop is selected within the target cluster. GFGD imposes the constraint that the target cluster must be surface-adjacent. GGFGD always has a longer path than GFGD, but, compared to Multipath Power-Control Transmission (MPT) (described in Section 2.5), both GGFGD and GFGD have longer paths because they take path loss, transmission delay, and residual energy level as part of the selection criteria as well, and, thus have higher average delay.

### 2.5. Energy Efficient Protocols

The Information-Carrying Routing Protocol (ICRP) [15] is an energy-efficient, real-time, scalable routing protocol. The source node checks the existing route to the destination when it has packets to send. If there is no existing route, it initiates a route formation process by broadcasting the data packet, which also carries the route discovery message. All the nodes that broadcast the data packet also maintain the reverse path. Route discovery is required every time the lifetime of the route expires. The Reliable and Energy Balanced Routing (REBAR) protocol [35] adaptively changes the broadcast domain to balance the energy consumption among the nodes. Nodes near the sink have a smaller radius to decrease their chances of being involved in routing, which increases their working life.

The Energy-Efficient Routing Protocol (EUROP) [36] reduces energy consumption by reducing the number of broadcast messages. EUROP uses pressure sensors in order to measure the depth, thus avoiding the need for hello messages. In the Power Efficient Routing (PER) protocol [37], the forwarding node is selected on the basis of distance, angle between the neighboring nodes, and residual energy. A node forwards the packet to the next node if the number of received duplicates does not exceed the limit. Compared to DBR, its energy consumption is lower. In the Energy-Efficient Depth-Based Routing (EEDBR) [38] protocol, the nodes share their depth and residual energy information with their neighbors. Packets are held by nodes for a certain time based on the residual energy, to avoid duplicates and balance the energy costs of packet forwarding. The nodes having less energy will wait longer. The Reliable Energy-Efficient Routing Protocol [39] takes into account link quality, physical distance, and residual energy. The three metrics are computed and shared by all the nodes.

The Delay-Aware Energy-Efficient Routing Protocol (DEEP) [40] is a 3-D routing protocol based on energy efficiency and collision rate. There is no handshake mechanism in DEEP. Relay nodes are selected on the basis of link quality and successful delivery ratio. A sequence number is used to discard duplicate packets. In the Channel Aware Routing Protocol (CARP) [41], the next forwarding node is selected on the basis of hop count and residual energy. Every node knows its hop distance from the sink node. The transmitting node broadcasts a PING packet to find the next forwarding node. If the hop count of the receiving node is less than the transmitting node, it sends back a PONG packet with its information.

The Energy Efficient and Collision Aware (EECA) [42] multipath routing algorithm is based on finding two collision-free routes using constrained and power adjusted flooding. Multipath power-control transmission (MPT) helps to deliver the packet with certain end-to-end packet error rate and minimum transmission power.

### 2.6. Reliable Data Delivery Protocols

The focus of this set of protocols is on reliability of data delivery. We present three examples. The Multipath Virtual Sink Architecture (MVSA) [43] uses multiple sinks, which connected together form a virtual sink. Instead of caching delay sensitive data, it is forwarded through multiple paths. This protocol increases the data delivery reliability at the cost of an increase in energy consumption. Efficient Data Delivery with Packet Cloning (EDDPC) [44] uses the same concept of multiple copies. However, it is a little bit different because it clones or copies selected data packets when forwarding them on the basis of link quality and channel conditions. 

The Resilient Routing Algorithm for Long-term Applications (RRALA) [45] is based on virtual circuit routing. Multihop connections are established in advance between each source and sink and each packet is associated with a particular connection. If a connection fails during the packet forwarding, another connection, established in advance, is used. 

### 2.7. Mobility Based Protocols

Mobile Delay-Tolerant Data Dolphin (DDD) [46] uses mobile nodes (Dolphins) to gather data from the stationary nodes. Mobicast Routing Protocol for Underwater Sensor Networks [47] is a 3D routing protocol based on mobile sinks, i.e., AUVs. Nodes close to an AUV form a 3D zone and the AUVs collect their data. For nodes to be ready, a sleeping node in the next zone is woken up in advance by the AUV.

## 3. Overview of Self Organized Fast Routing Protocol (SOFRP)

This section provides an overview of the proposed protocol, prior to the detailed description in Section 4. Our proposal is based on the automatic formation of predefined routing paths to forward the data packet from each node to the sink, without the use of additional measurement devices, such as pressure sensors. However, it is assumed that nodes are placed in a specific formation rather than deployed randomly. For easier reference of the acronyms used, they can be looked up in Section 5, where Table 1 lists the acronyms used for names of messages and Table 2 contains the acronyms used to denote mathematical parameters.

### 3.1. Network Topology

It is well understood that the larger the number of sensor nodes deployed in an area the more complete the area monitoring and characterization. Therefore, deploying the large number of sensors in a grid or hexagonal form is preferred for more accurate monitoring. This relationship between nodes density and information accuracy is valid for all kind of monitoring systems, whether terrestrial or underwater, wired or wireless. However, such high accuracy is not always required for all kind of monitoring systems. High density deployment of the nodes has a substantial effect on the cost. Compared to terrestrial monitoring systems, the costs of the underwater sensor nodes and their deployment are very high, especially in deep waters [48]. Therefore, monitoring systems having a low density of sensor nodes are preferred (whenever high accuracy is not required) to reduce the costs. Underwater monitoring systems such as an early warning system for tsunamis [49] and volcanic eruption monitoring [50,51] will perform well enough with low density sensor networks. In such cases, radial topology, with a larger density at the core, is more suitable to reduce the costs of the monitoring system.

**Figure 2:**
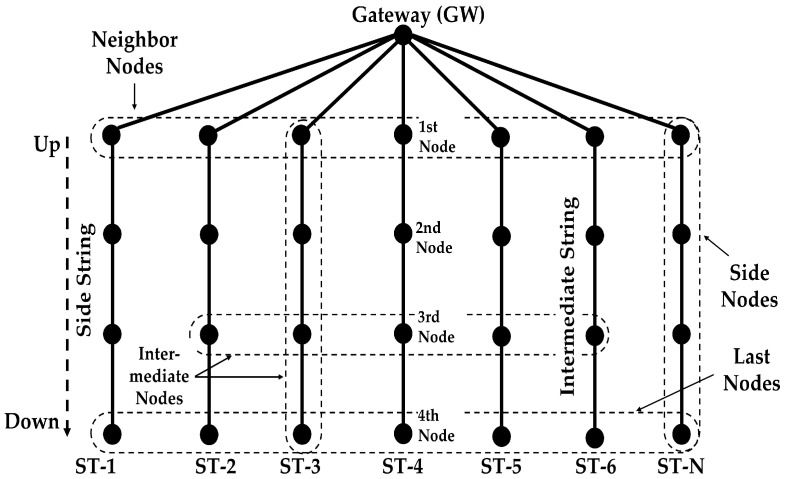
Network Topology of N Strings.

Our protocol is proposed for a kind of hybrid radial/linear topology as shown in Figure 2. The reason is that this particular topology is very much suited for monitoring river plumes, where sediments are carried away from a river mouth into the sea, whose scattering follows a triangular pattern, moving sideways with the sea current. It can also be used for monitoring oil spills, coral reefs and habitats along the coastal area. In general, the topology serves to monitor an area and collect all the information in a single point that connects the network to the outside world.

We consider that the network contains an indeterminate number of nodes and a gateway (GW), with *N* radii, where *N* = {1,2, …, n}. From now on, we will use the name of string for the radial lines. The strings are shown in vertically in Figure 2, but they can be organized as radii departing from the gateway in all directions, distributed over the circle (2D) or the sphere (3D). This topology fits any 3D region shape when only one sink node is possible. 

In terms of interference, the worst case is when adjacent strings are parallel to each other, and this will be the case considered. For the purpose of the analysis in this paper, we have considered an equal distance among the nodes in each radius. Varying distances can be considered by setting an appropriate guard time in the transmissions, within a limit. Finally, we consider that nodes adapt their transmission power so that only neighbor nodes can be reached. Thus, nodes that are two or more hops away from the source do not hear its transmissions.

**Figure 3:**
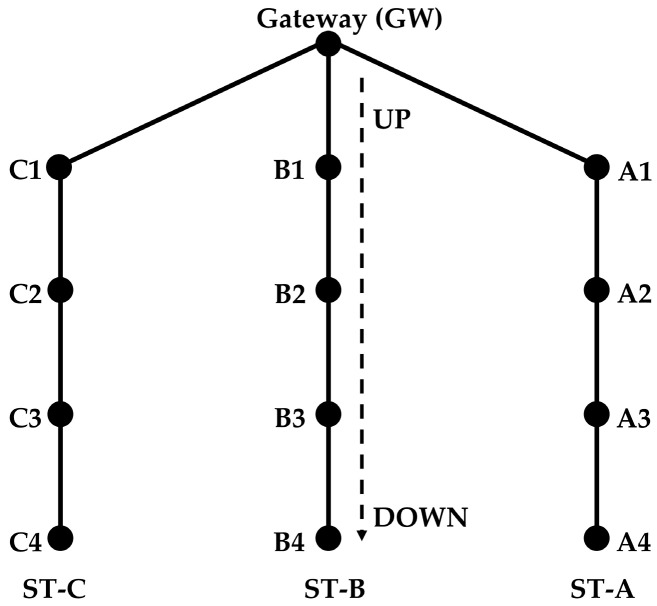
Example of network with three Strings.

This protocol is designed for an arbitrary number of strings. Along the description, we have mainly considered a network of three strings (Figure 3) for easier understanding, but the algorithm is designed for any number of strings. 

### 3.2. Proposed Protocol Overview

The gateway is the node at the surface and relays the data of the other nodes to the terrestrial network. It is also the controlling node of the network. The gateway receives data only from its neighbors. For example, nodes A1, A2, A3, and A4 in Figure 3 form a routing path. The nodes in each string forward the data of only those nodes that are above or below them in the same string. When the nodes send data to the gateway, they forward the data to the node above. When the nodes forward control packets from the gateway, they send the packets to the nodes below them. 

For explanation purposes, the outer strings are called side strings and all the inner strings are called intermediate strings. Nodes in the outer strings are called side nodes and nodes in the intermediate strings are called intermediate nodes. The nodes that are farther away from the gateway are called down nodes. The transmission range of a node is limited to its adjacent nodes, using power control adaptation according to the distance between nodes. 

The routing path formed in this way presents two advantages. First, it creates the shortest path between the sender node and the gateway, which minimizes the delay in terms of the number of hops. If a node were allowed to forward the packet to the adjacent node on the same layer then it would increase the total number of hops, which would increase the delay. Second, it keeps the traffic balance among the gateway neighbor and avoids earlier failure of a gateway neighbor due to excessive transmission.

Initially the nodes have no knowledge about the existence of each other on the network. In order to form the routing path, they need to exchange certain information. The gateway starts the routing configuration process by broadcasting a packet to find its neighbors. The neighbors of the gateway (Figure 3) first look for the nodes in their neighborhood that are at the same level (horizontally). This is the set of nodes that is one hop from the gateway, and each of them is the first node in one string (see Section 4.1) 

It is possible that the packets broadcast by the gateway do not reach some or any of its neighbor nodes due to channel noise. If any of the neighbor nodes fails to receive the broadcast packet, then that node and all the nodes below it will not be able to create the routing path. To address this issue, we have designed the protocol using cross-layer techniques, merging some steps of the route creation process with the medium access control layer. Initially, both the neighbor nodes and the gateway try to discover each other. This is achieved by means of beacon packets, transmitted periodically by each node. When the gateway receives a beacon packet from any of its neighbor nodes it will wait for a specific period and then send an enquiry packet to find its immediate neighbors. 

Once the gateway knows the first layer of neighbor nodes, it sends a Route Request (RR) packet to form the strings. The neighbor nodes and those nodes below them forward the request packet, hop by hop, until it reaches the end of each string. Then starts the response process, which sends information about the completion of the string formation. The unique string ID is used by the data packets to send data to the gateway on the corresponding route. When a node receives the data packet, it compares its string ID with the string ID field in the packet header. If both string IDs are the same, then the node forwards the data packet; otherwise, it discards it. Step-by-step detail of the string formation process is given in Section 4.2.

To avoid collisions, the nodes each transmit routing packets at different times. A node will randomly select a timeslot (TS) from a set of *k* timeslots. The length of a timeslot is equal to the sum of the propagation delay, the transmission delay and a guard time. The duration of *k* timeslots is called wait period (WP). Assume that the maximum distance between two nodes is 1000 m. If the maximum packet size is 16 bytes and the channel data rate is 5 kbps, then the time interval is 0.666 + 0.0256 = 0.6916 s (approx.). If we consider a number of 40 timeslots, they would start at instants 0, 0.6916, 1.3832, 2.0748, 2.766, …, 26.972 s. The total of 40 timeslots would last for 27.664 s, which would be the WP value. In this configuration, the probability of two nodes selecting the same timeslot out of 40 timeslots is 2.5%. No matter how large the number of timeslots, the chances of collision are always there. 

Different types of transmission modes have been used to optimally achieve delivery reliability and energy efficiency. There are four modes of transmission named mode1, mode2, mode3 and mode4.

- mode1: the packet is transmitted once without any acknowledgement.
- mode2: the packet is transmitted three consecutive times without any acknowledgement. The interval between transmissions is equal to one timeslot (TS).
- mode3: all the candidate nodes send a packet within a period equal to the wait period. Then, acknowledgements are sent during the next WP. Hence, the total time for sending a packet and receiving its acknowledgment is 2*WP. This process of sending a packet and receiving its acknowledgment may repeat two more times in case of packet loss. Therefore, the overall period for the three transmissions remains fixed (which is 6*WP) whether the number of transmissions is one, two or three.
- mode4: this mode is a little bit different from mode3 in terms of packet retransmissions. In mode4, the packet and its acknowledgement are transmitted three times, even if the packet was successfully delivered in the first or the second transmission.

The main novelties of this protocol are:

(a) It is designed to recreate the physical topology in the logical routing tables, creating the shortest possible paths,
(b) The algorithm uses the knowledge of the topology to avoid collisions,
(c) It helps to balance traffic among nodes that are the same distance from the gateway,
(d) Our proposal self-adapts to different network sizes and number of nodes.

Together, these characteristics help to save as much energy as possible, and minimize the cost, in time and computational effort, of forwarding a packet.

## 4. Routing Path Formation

We can divide the formation of routing paths in two phases, which are described in detail in this section.

➢ Phase 1: Search for gateway neighbors
➢ Phase 2: String formation

### 4.1. Phase 1: Search for Gateway Neighbors

**Figure 4:**
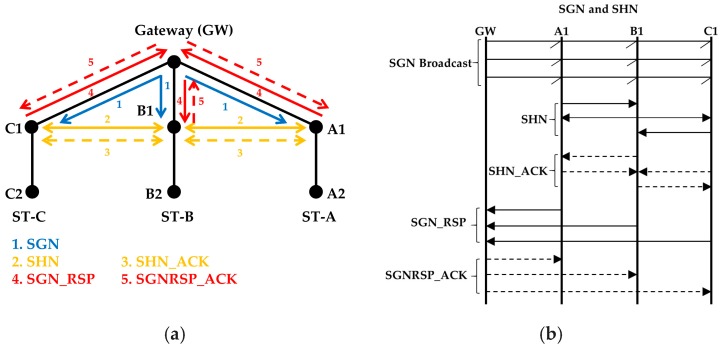
(**a**) Transmission sequence of SGN, SHN, SHN_ACK, SGN_RSP and SGNRSP_ACK between the gateway and the neighbors, (**b**) The same transmission sequence is also shown in message sequence diagram.

As we mentioned earlier, in Section 3, the gateway is the controlling node of the network and it can only communicate with its neighbor nodes, which are at one hop distance. The gateway is responsible for starting the process of forming the strings. Since, initially, the gateway does not know about its neighbor nodes, it finds them first. This phase follows these steps:

1. Initially, all the nodes transmit a beacon packet, using mode1, at random time slots to inform of their presence. Other than the gateway, all other nodes ignore the packet.
2. When the gateway receives a beacon packet from any of its neighbor nodes it will wait for a WP. This avoids a possible collision between the SGN packet (see next step) and a beacon packet from another node.
3. The gateway broadcasts the first Search Gateway Neighbor (SGN) packet to find its neighbors (see 1 in Figure 4a). Altogether, the gateway sends the SGN packet using mode2 (three consecutive transmissions) (Figure 4b). The SGN packets carry a sequence number.
4. The nodes that receive the first SGN packet wait to receive two more SGN packets before they start finding horizontal neighbor nodes. Suppose that a node fails to receive the first two SGN packets but receives the third SGN packet. This node does not wait for two more SGN packets because it knows from the sequence number that this is the third SGN packet.
5. After receiving the SGN packet(s), each neighbor broadcasts a Search Horizontal Neighbors (SHN) packet to find its horizontal neighbors (see 2 in Figure 4a) using mode3. Each node selects a random timeslot for transmission.
6. The nodes that received a SGN packet earlier, send an SHN acknowledgment (SHN_ACK) packet. (see 3 in Figure 4a). The SHN_ACK packets are transmitted using mode3 after all the neighbor nodes have finished sending their SHN packets. The nodes in the lower layer receive the SHN packets as well but they ignore them because they did not previously receive the SGN packet from the gateway.
7. When the waiting time for SHN_ACK packets finishes, each neighbor node sends an SGN response (SGN_RSP) packet to the gateway (see 4 in Figure 4a), using mode3. The SGN_RSP packet includes information on the neighbors of the sender. When all responses are received, the gateway will know its neighbors and can determine their relative positions by the horizontal neighbor information of each node.
8. The gateway sends back an SGN_RSP acknowledgement (SGNRSP_ACK) packet at a random time, using mode3 as well.
9. To form a string, the gateway assigns a unique string identification (ST_ID) number to each neighbor. The nodes below each gateway neighbor will be part of that straight hop-by-hop forwarding path.

We can explain mode3 in more detail with the help of the SHN and SHN_ACK packets. If the SHN_ACK packet is not received by a node, it will retransmit the SHN request. If the SHN_ACK packet is still not received, then the sender will transmit the SHN packet a third and last time. The next step, transmission of the SGN_RSP packets from the neighbors, will start after six WPs, which is the time required for a sequence of three iterations of SHN and SHN_ACK packets. However, during this period the SHN packet will be retransmitted only if the SHN_ACK packet is not received.

### 4.2. Phase 2: String Formation

**Figure 5:**
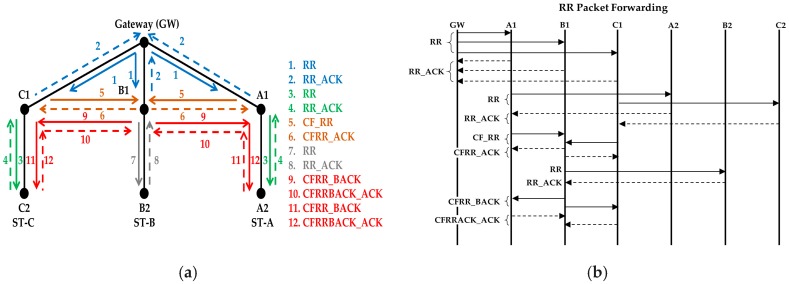
(**a**) Transmission sequence of RR, RR_ACK between the gateway and the neighbors and transmission of RR, RR_ACK, CF_RR, CFRR_ACK, CFRR_BACK and CFRRBACK_ACK between the layers and the adjacent nodes (**b**) The same transmission sequence is also shown in message sequence diagram.

**Figure 6:**
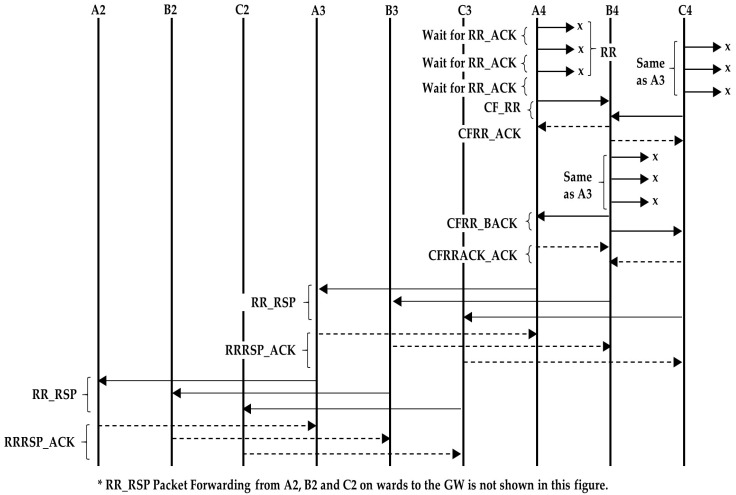
Propagation of the RR_RSP message.

In this phase, the routing paths will be formed. The gateway allocates a string identification (ST_ID) to each neighbor node. The gateway neighbor nodes are called the first nodes in the strings, the next nodes below them are called second nodes, and so on (see Figure 2). The process will be completed layer by layer. That is, first of all, the first nodes in the strings will identify the second nodes in the strings, then the second nodes will identify the third nodes, and this process will continue until the end of each string. In each layer, the identification of the nodes will start from the side nodes and will continue inwards through the intermediate nodes. Step-by-step detail of the procedure is given below:

1. First, Route Request (RR) packets are sent from the gateway to each of its neighbors (see 1 in Figure 5a) using mode4. The RR packet contains the IDs of all the neighbors and their respective string IDs. When a neighbor receives the RR packet for the first time it checks the list of node IDs to know its assigned string ID.
2. The neighbor nodes will send the RR acknowledgement (RR_ACK) using mode4 as well.
3. After that, first the side nodes in the string will forward the RR packet (see 3 in Figure 5a), now using mode3. The RR packet will be received by all adjacent nodes within range. These RR packets contain the node IDs with their corresponding string IDs and packet sequence number.
4. When a node receives the RR packet, it records the ID of the sender and its respective ST_ID. The gateway discards any RR packets it receives from its neighbors. The nodes that already have received the RR packet also discard the RR packet.
5. Only the node below in the string will reply with the RR_ACK packet, which will contain the sender ID and the ST_ID. They use mode3.
6. The node that receives the RR_ACK packet as a reply to a previous RR packet transmission records the node and the string IDs in its routing table. This way each node has complete information about the nodes above and below it in the string.
7. Now, the side nodes of the first layer send a Clear to Forward RR (CF_RR) packet (see 5 in Figure 5a) to their adjacent horizontal nodes (first nodes of the intermediate strings) using mode3.
8. The intermediate nodes send a CF_RR acknowledgement (CFRR_ACK) packet using mode3.
9. The process of sending RR and CF_RR will continue repetitively as described above until all the first layer nodes in all the strings have sent an RR packet to the second layer nodes.
10. When the last intermediate node in the first layer sends the CF_RR packet, it will not receive the CFRR_ACK reply because it is the last intermediate node. It will retransmit the CF_RR packet two more times and then it will decide to send a CFRR_BACK packet.
11. The CFRR_BACK packet will travel from this last intermediate node in the first layer to the side nodes in the second layer (see 9 in Figure 5a). CFRR_BACK and CFRRBACK_ACK packets will also be transmitted using mode3.
12. When the node of the second layer in the side string receives the CFRR_BACK packet, it starts the process of forwarding the RR packet to the third layer node.
13. Assuming that the number of nodes in all the strings is the same, the last nodes of the side strings will transmit the RR packet three times, but will not get any acknowledgement. Hence, they will conclude that they are the last nodes and will send the CF_RR packet to the intermediate nodes, with information that they are the last nodes. The intermediate nodes also transmit an RR packet, using mode3, and then send a CFRR_BACK packet to the side nodes informing that it is the last node of the string as well. At this stage, all the last nodes randomly select a time slot and send the RR_RSP response (see Figure 6).
14. When the RR_RSP packets reach the gateway neighbor nodes, they forward the packet to the gateway. Each neighbor node waits to receive the RR_RSPACK packet from the gateway after transmitting the RR_RSP. After receiving the RR_RSP packet, the gateway knows that the routing path formation is complete for that string. RR_RSP and RRRSP_ACK are sent using mode3.
15. The gateway waits for a threshold period to receive the RR_RSP from all of its nodes. If the RR_RSP packets are not received within that threshold period, the gateway starts a new process of finding the path by sending a new RR packet. A packet sequence number is used so that the nodes know that this RR packet is different from the old one.

In case the number of nodes in the side strings is smaller than the number of nodes in the intermediate strings and the side strings have reached the end, the side nodes will send their CF_RR packets to the intermediate nodes before sending the RR response (RR_RSP) packet. When the intermediate nodes receive the CF_RR packet, they will transmit the RR packet and wait for the acknowledgement. Once the intermediate nodes finish the process, they send the Start Response (ST_RSP) packet to the side nodes to inform them that they can initiate the transmission of the RR_RSP packet. In this case, the intermediate nodes send a ST_RSP packet to the side nodes instead of the regular CFRR_BACK packet. The intermediate nodes need to know in which occasions to send the ST_RSP packet. For that, the side nodes send information to the intermediate nodes, in the CF_RR packet, indicating that they are the last nodes and they are waiting for the ST_RSP packet to send the RR_RSP response packet. The ST_RSP and RR_RSP packets are also sent using mode3. 

Similarly, if the intermediate strings end before the side strings, the intermediate nodes wait for the ST_RSP packet before sending the RR_RSP packet. When the last intermediate nodes send the CF_RR packet to the side nodes, they also indicate that they are the last node in the string. The side nodes send the ST_RSP response to the intermediate nodes after finishing the process of finding the next nodes. The side nodes also know that the intermediate strings have ended and when they forward the RR packets, they neither send CF_RR to the intermediate node nor wait for the CFRR_BACK from the intermediate nodes. The side nodes simply forward the RR packet to the next node in the string after sending RR_ACK to the node from which they received the RR packet. 

If a node was up when the RR packet was forwarded but is down when the RR_RSP packet is sent, then the RR_RSP packet from that particular string will not reach the gateway. In that case, the gateway will send the RR packet again for that string only. In case more than one node is down in multiple strings, the gateway starts the process of route finding for all the nodes again.

### 4.3. Adaptation for Hexagonal and Grid Topologies

**Figure 7:**
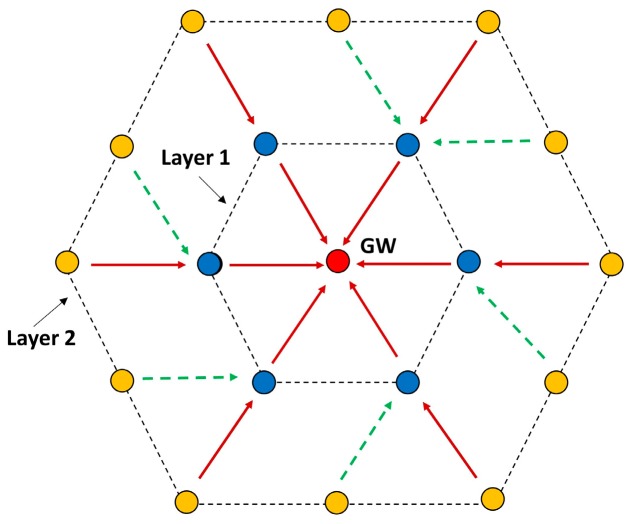
Hexagonal Topology.

This protocol may also work for a hexagonal topology. When the gateway receives the SGN_RSP packet (see Section 4.1) from the neighbors, it can easily deduce that the neighbors are located in a ring around it, because all gateway neighbors indicate that they have two neighbors each. When the gateway sends the RR packet (see Section 4.2) to the neighbors, it will set the ring field in the header to true. This will allow a node to form multiple paths with multiple nodes. Each node in a layer may form paths with 1, 2 or 3 nodes in the next layer as shown in Figure 7. 

The neighbor nodes send the RR packet at randomly selected timeslots. The first RR packet will be received by three next layer nodes and they will form the path with the RR-sending neighbor (shown in Figure 7). The rest of the neighbor nodes form two paths with the next layer nodes, except the last node, which will form only one path. Thus, the resulting paths may be unbalanced, but this can be solved either by choosing one of the possible paths or by using all paths in a round-robin fashion, which would balance the energy consumption among the forwarding nodes.

**Figure 8:**
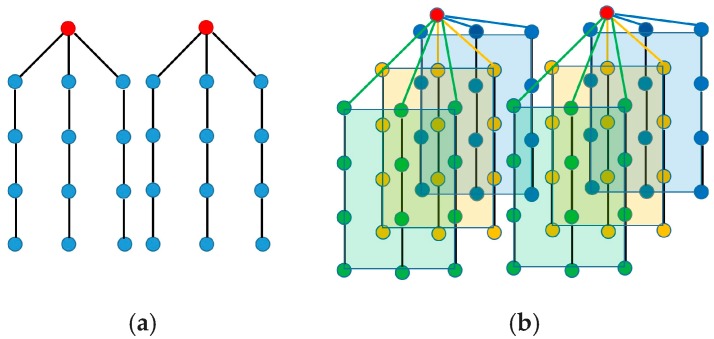
(**a**) 2D Grid; (**b**) 3D Grid.

In the case of a grid topology, the network can be split in several sections as shown in Figure 8, each having its own sink (gateway). Each section then can be considered to have the topology shown in Section 3.1 (Figure 2 and Figure 3).

### 4.4. String Formation in Case of Node Failure 

Let us analyze the situation in which one of the nodes is down either at the time of initialization or after the initialization. These two situations and their solutions are discussed below in Scenario 1 and Scenario 2. 

#### 4.4.1. Scenario 1: A Node is down at the Time of Routing Initialization. 

**Figure 9:**
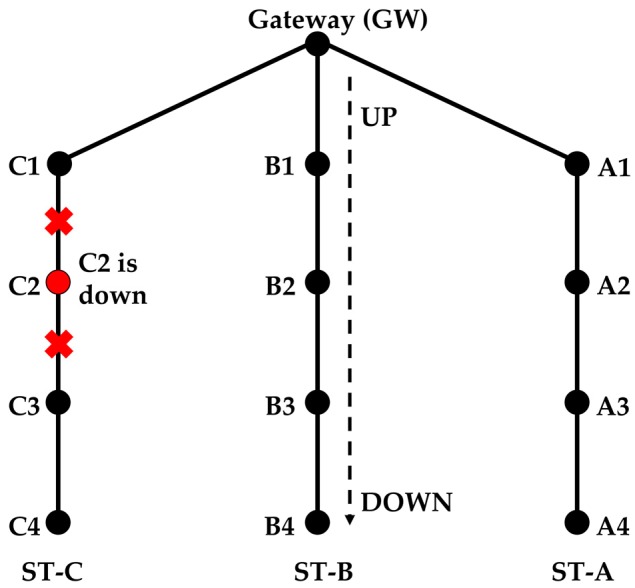
A node is down at the time of routing initialization.

The down node can be one of the side or intermediate strings. Consider a network with three strings (see Figure 9) and assume that C2 is down during the routing initialization. C1 sends the RR packet three times and finally thinks that there are no more nodes in the string. It will send the RR_RSP response as mentioned in Section 4.2 paragraph (13). In this case, the RR_RSP is sent not because the string has ended, but because a node failure has occurred. When C1 sends the CF_RR to the intermediate node, it also indicates that it is the last node in the string (although this is not true). Once the intermediate node finishes sending and receiving the RR and RR_ACK packets, respectively, it sends a CFRR_BACK to the side nodes.

Continuing to form a string in this way creates a problem. Suppose that up to C1, ST-C is formed. When B3 forwards the RR packet, it is supposed to receive a RR_ACK from B4 only. But since previously, C3 failed to become part of ST-C, it also responds to B3. Upon receiving two RR_ACK packets, B3 understands that either B4 or C3 is not part of its string. However at this stage, B3 cannot decide which one.

**Solution**

**Figure 10:**
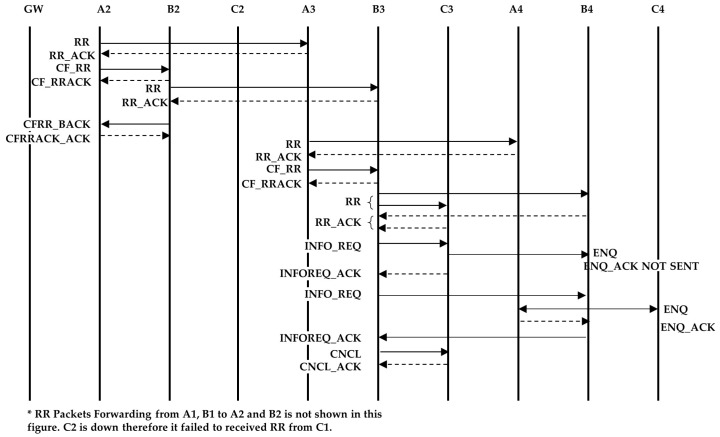
Sequence of messages to determine the correct node for string formation.

Under this situation, B3 will not send a normal CF_RR packet instead, it sends an information request (INFO_REQ) packet, which asks nodes B4 and C3 (one by one) to send an enquiry packet (ENQ), and waits for the INFO_REQ acknowledgement (INFOREQ_ACK) packet (Figure 10). If the INFOREQ_ACK packet is not received after three attempts, then the node waits for a certain time and retries two more times. Assume B3 first asks C3 to send the ENQ packet. When C3 sends the ENQ packet, only the nodes that are already part of a string send an enquiry acknowledgement (ENQ_ACK) packet. Hence, C3 does not receive the ENQ_ACK packet from C4, as it is not part of a string yet (B3 does not send an ENQ_ACK packet because it originally asked C3 to send an ENQ packet). C3 sends an INFOREQ_ACK packet to B3 indicating that it did not receive any acknowledgement. Similarly, B3 asks B4 to send an ENQ packet. B4 will receive the ENQ_ACK packet from A4. B4 provides that information to B3 in the INFREQ_ACK packet. Now, B3 knows that B4 is the intermediate node, because it received the ENQ_ACK packet. B3 sends a cancel (CNCL) packet to C3, which tells it that it has wrongly become part of string ST-B and it should initiate forming a new string. B3 has not sent the CFRR_BACK at this point yet. To form string ST-D, C3 will send a RR packet to C4 and, after receiving the acknowledgement, it will send a CNCL_ACK (acknowledgement) packet to B3. Upon receiving the CNCL_ACK packet, B3 will send a CFRR_BACK packet to C3 and A3 as described before. 

When string ST-D reaches the end node, it starts sending the RR_RSP packet. When the RR_RSP packet reaches C3 it knows that it initiated a new string and the node above is down, therefore it will not forward the RR_RSP packet. In order to send data of ST-D via strings ST-B and ST-C we use the same method of route discovery as mentioned in Scenario 2. This route discovery process starts after the completion of the routing initialization process. 

#### 4.4.2. Scenario 2: What will Happen If a Node in a String Goes down after the Initialization?

A mechanism is required to forward the packets in case one of the nodes of a string is down. First, the nodes above and below the failed node in that string should be informed about the failure so they can make an alternative path. Timeout threshold and beacon signals are used to address this issue. If a node does not transmit a packet for a certain period, i.e., 10 WPs, then it should transmit a beacon packet to indicate that it is still alive. The nodes should also expect to receive a data or a beacon packet from the nodes above and below them within the threshold period. If a node fails to transmit data or beacon packets, then the nodes above and below in the string conclude that the node has died and start the process of finding the alternative path. 

**Solution**

**Figure 11:**
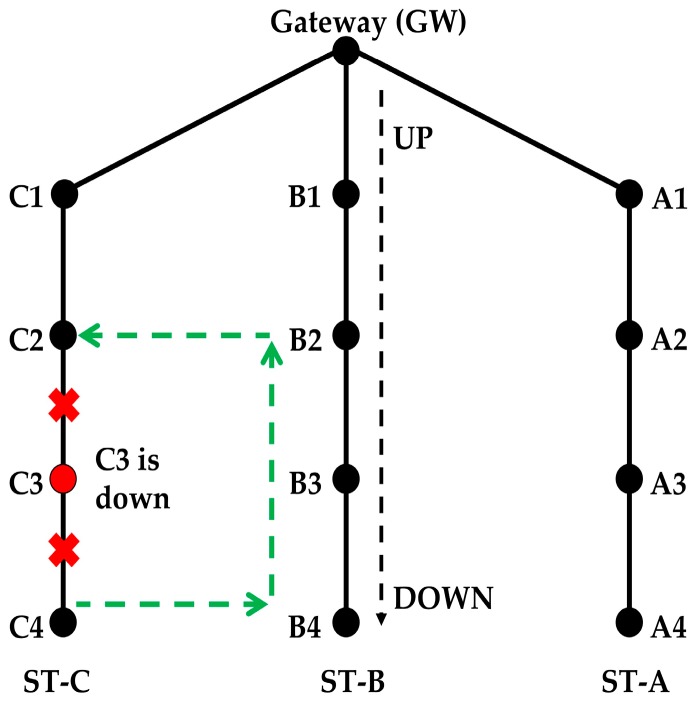
Alternative path when a node fails after the routing paths have been formed.

The only solution is that the nodes of the adjacent strings forward the packet. One such alternative path is shown in Figure 11. In this example, it is assumed that node C3 has gone down and the data of C4 is forwarded to C2 via B4, B3 and B2.

In this way, the traffic load is balanced as much as possible between the nodes of the two strings. If all the packets of all the nodes below the failed node in string ST-C are forwarded via string ST-B only, then nodes of ST-B will be overloaded. A new ST_ID is used for the packets transmitted by C4 to B4. B2 should forward packets of its own string with a different string ID than packets of ST-C. This way, B1 forwards data packets of its own string only in order to create a balanced energy consumption. Suppose that B2 forwards a packet of ST-C with a different string ID, say ST-D. The forwarded packet reaches both B1 and C2, but only C2 accepts the packet. When C2 receives packets from B2 with string ID ST-D, it changes the string ID to ST-C before it forwards the packet to C1. Therefore, nodes B2, C2, B4, and C4 handle two different string IDs. When C4 sends or receives data from the node below it (if a node is there), it uses string ID ST-C, but when it sends data to B4 or receives data from B4, it uses string ID ST-D. Two IDs are used by all nodes that form the alternative path.

The node below the failed node initiates the new route-finding process. It broadcasts an RR packet with a new string ID and its existing string ID. Nodes below the initiating node in the same string ignore the RR packet. The nodes in the adjacent string(s) receive the RR packet and recognize that it is sent from the adjacent string. Knowing that, they send back the acknowledgement.

Suppose C4 initiates the new route discovery process. It broadcasts an RR packet, which is received by B4 and it sends back the acknowledgement. 

Now B4 will forward the RR packet, which will be received by B3 and A4. A mechanism is required such that only the node above (B3 in this case) accepts the forwarded RR packet. For that, B4 adds information in the RR packet, which tells the receiving nodes that this RR packet is not generated by the sending node. When A4 receives the RR packet and it learns that B4 simply forwarded the RR packet of another string, it will not send the acknowledgement to B4. Only B3 will send the acknowledgement to B4. B3 will also forward the RR packet to B2 and it will become part of this new string. 

Now, B2 forwards the RR packet, which is received by nodes A2, B1, and C2. Node A2 will ignore the received RR packet because of the different string ID. B1 and C2 both send the acknowledgment to B2. B2 decides to make a path with C2 rather than B1 because it gets a reply from C2, which has the same string ID as C4. B2 sends a packet to B1 to inform that it is not part of the ST-D, and another packet to C2 to inform that it is now part of ST-D as well. This way, a new route is established between nodes C4 → B4 →B3 → B2 → C2.

In case one of the intermediate string nodes fails, the procedure of the new path selection will be almost the same with one exception. Suppose B2 is the failed node and B3 is the initiating node. B3 generates an RR packet, which is received by both A3 and C3. Both A3 and C3 send acknowledgment to B3 along with their energy level information, and B3 chooses the node that has the higher energy level. 

### 4.5. Other Issues

#### Routing Path Periodic Update Process 

The routing initialization process needs to be executed from time to time in order to eliminate any change in the routing path due to movement, failure or addition of nodes. Before the process is initiated by the gateway, the data communication from the nodes must be stopped, otherwise collisions may occur. For that, the gateway broadcasts an alert packet to all of its neighbor nodes, and throughout the strings. All the nodes that receive the alert send back acknowledgement to the gateway. The gateway already knows about all the nodes in the network. If it fails to receive the acknowledgment of the alert packet from any of the nodes, it sends the alert packet again after waiting for the maximum possible delay. If the gateway still fails to get the acknowledgement, it assumes that the node or nodes are down and starts the routing path update process using the same initial configuration process described above.

### 4.6. Data Packet Forwarding

When a node receives a data packet to be forwarded, it looks into the routing table and compares the string ID of the received data packet with its own string ID. If the string ID of the packet matches with the node’s string ID, it forwards the packet. 

## 5. Packet Format

**Figure 12:**
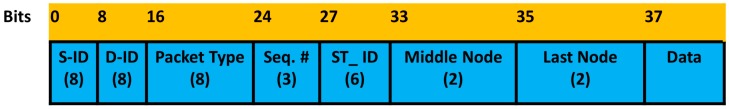
Packet Header Format.

A packet is composed of a header and a payload. There are seven header fields, namely S-ID, D-ID, Packet Type, Seq#, ST_ID, Middle Node, and Last Node. The total length of the packet header is 37 bits. The packet header format is shown in Figure 12. The first two fields are the source node ID (S-ID) and the destination node ID (D-ID). The D-ID will be FF for broadcast communication. The packet type field helps the receiving node to identify the type of the packet. Total length of the packet type field is eight bits. Seven bits out of eight bits are used for the unique code for each packet type (shown in Table 1) and the last bit (LSB) is used to indicate the ring topology. LSB “1” indicates that the topology is the ring topology. In case of multiple transmissions of the same packet, such as the RR packet, the three-bit sequence number (Seq. #) is used to identify duplicate packets. The string identification (ST_ID) is represented by six bits. With the Middle Node field, the gateway informs its neighbors, in the RR packet, whether they are side nodes or intermediate nodes. The Last Node field is used by the last node in a string when it sends a CF_RR or a CFRR_BACK packet. The list of packet names, their description, and code is given in Table 1. 

## 6. Performance Analysis

We have developed a mathematical model to calculate the convergence delay, that is, how long it would take to create the routing paths, and compared it with the simulated convergence delay. The calculations are carried out with the help of MAPLE^®^. The list of the symbols used in the following equations is given in Table 2. 

For the mathematical analysis, we will assumed that the number of nodes in all the strings is the same. First, we will calculate the duration of one timeslot. This is the amount of time reserved for a packet to reach from one node to another. This value depends on two factors: the packet size and the propagation delay. Equation (1) gives the size of the packets in bits. Equations (2) and (3) calculate the packet delay and the propagation delay respectively. Equation (4) calculates the guard time to take into account variations in propagation delay. The guard time is 5% of the total of the packet transmission delay and the propagation delay. Equation (5) adds packet transmission delay, propagation delay and guard time to obtain the duration for one timeslot.

PS = 37 + 8 * ND,(1)

PKD = PS/DR,(2)

PROD = DT/SPS,(3)

GT = (PKD + PROD) * 0.05,(4)

TS = PKD + PROD + GT,(5)

Next, we developed the equations to calculate how long the gateway takes to find its neighbor nodes plus the time it takes the neighbor nodes to find their horizontal neighbors. Equation (6) calculates the length of the wait period. As mentioned in Section 4.1, the gateway waits one WP before transmitting SGN packets in order to avoid a collision with any of its neighbors, which might be broadcasting a beacon packet (see Equation (7)). Then the gateway sends an SGN packet three times. Equations (8) and (9) give the delay for the SHN and SHN_ACK packets and for the SGNRSP and SGNRSP_ACK packets, respectively

WP = TS * (TTS − 1),(6)

D_SGN_ = WP + 3 * TS, (7)

D_SHN_ = 6 * WP, (8)

D_SGNRSP_ = 6 * WP (9)

The delay for the pairs of SHN and SHN_ACK packets, RR and RR_ACK packets from the gateway to the neighbor nodes and RR_RSP/RRRSP_ACK packets is shown in Equation (10). Two WPs are required for packet sending and receiving and, since the packets will be sent three times, total WPs will be 2 * 3 = 6.

D1 = 6 * WP(10)

The delay for the pairs of RR and RR_ACK, CF_RR and CFRR_ACK and CFRR_BACK and CFRRBACK_ACK packets is shown in Equation (11):

D2 = 3 * D1(11)

The process to send the RR and CFRR packets proceeds from both sides of the network to the intermediate node. The number of intermediate nodes is even if the number of strings is even, otherwise there will be an odd number of intermediate nodes. Merging both scenarios, the process is repeated half times the number of strings and the result is rounded up to the next integer for an odd number of strings (Equation (12)). As mentioned in Section 4.2 the RR forwarding process is completed layer by layer and this process is repeated (ND-1) times (Equation (13)).

RPH = ceiling(ST/2),(12)

RPV = ND − 1,(13)

If we add all the delays calculated up to this point, we obtain the total delay for RR packet forwarding from the gateway to all the nodes in the network (see Equation (14)). After the RR packet has been forwarded, the last nodes in the strings start sending the response. Since all the nodes of each layer send an RR_RSP packet at randomly selected timeslots, we simply multiply the delay for one pair of RR_RSP/RRRSP_ACK packets (Equation (15)) by the number of nodes in the strings. Finally, to get the total delay, we add Equations (14) and (15) to get Equation (16). Equation (17) is the expansion of Equation (16).

D_RR_ALL_ = RPV * (RPH * (D2)) + D1, (14)

D_RR_RSP_ALL_ = ND * D1, (15)

D_TOTAL_ = D_RR_ALL_ + D_RR_RSP_ALL_, (16)

D_TOTAL_ = ceiling(ST/2) * ((ND-1) * (D2)) + D1 + ND * D1 (17)

**Figure 13:**
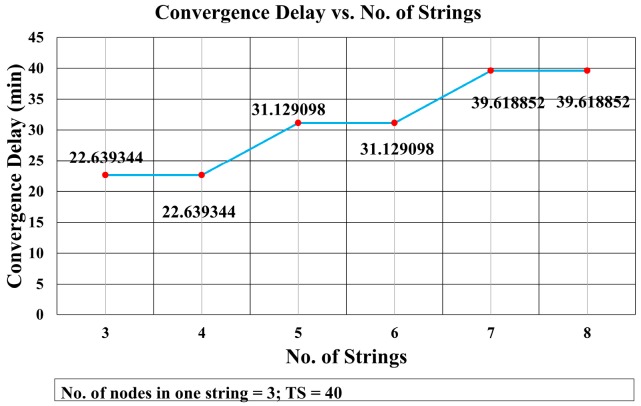
Convergence Delay vs. No. of Strings.

**Figure 14:**
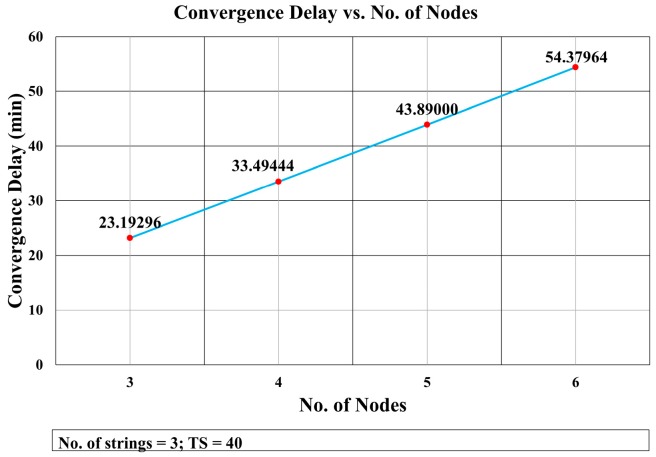
Convergence Delay vs. No. of Nodes.

This mathematical analysis has been validated via simulation, as described in the next section. Several cases have been simulated to assess the convergence delay, such as when the number of strings and nodes varies (see Figure 13 and Figure 14, and Tables 4 and 5), and for a varying number of timeslots in the wait period (Figure 19). A comparison between the values obtained via the mathematical equation, Equation (17), and via the simulations is shown in Figure 19. As it can be seen, there is an almost perfect agreement between both methods.

## 7. Computer Simulation Results

The performance of the protocol is analyzed in terms of three parameters, i.e., convergence delay, probability of collision between packets and number of retransmitted packets in case of packet loss. Convergence delay is defined as the total time required to form the routing paths, which starts with the transmission of the beacon packets and ends when the gateway successfully receives the RR_RSP packets. 

For the simulations, we have assumed a topology with the structure shown in Figure 3. The distance between the gateway and its neighbor nodes is set at 500 m. Similarly, the distance between two adjacent nodes in a string is also set at 500 m. The number of nodes in all the strings is the same. Propagation speed is taken as 1500 m/s. The data rate is 5000 bits per second and the packet size is calculated using Equation (1). The transmission power considered for the simulations is 18 W (a value taken from Reference [52]), and the BER is 10^−6^. The simulation parameters are summarized in Table 3. All these values are realistic in UW communication networks. The simulation tool is a proprietary MATLAB^®^-based simulator. 

First, we analyze the effect of increasing the number of strings on the convergence delay. Figure 13 shows the results of a simulation in which there are three nodes in each string, but the number of strings varies. It shows that the convergence delay remains the same when the number of strings increases from odd to even, as for example from three to four or from five to six. This is due to the fact that the number of transmissions of RR packets and CF_RR packets remains the same when the number of strings increases from odd to the next even number. Hence, the delay remains the same for a number of strings (1, 2), (3, 4), (5, 6), and so on. The difference of delay between string 4 and 5 is 8.489754 min. Similarly, the difference between strings 6 and 7 is also 8.489754 min.

The effect of increasing the number of nodes in a string is depicted in Figure 14. It shows that the convergence delay increased linearly with the number of nodes. This was as expected because the delay incurred by each layer is almost constant. The delay increased as the number of nodes grew due to the increase in the packet size. The difference in the packet delay transmission was constant (0.0016 s) for consecutive increases in the number of nodes (see Table 4). The impact of augmenting the network in one node per string in the total convergence delay was 0.091728 (see Table 5).

**Figure 15:**
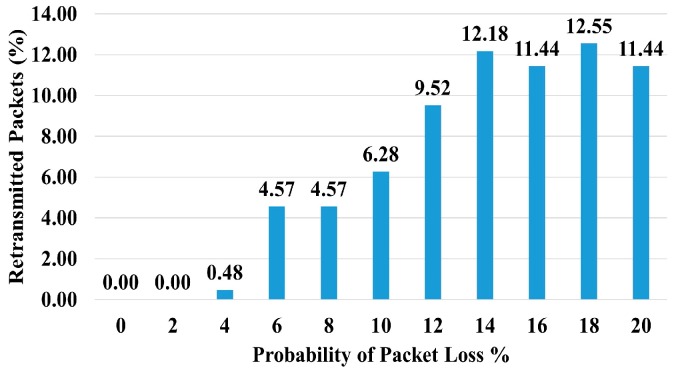
Number of Retransmitted Packets vs. Probability of Packet Loss.

We have analyzed the number of packet retransmissions when the probability of packet loss increases from 0 to 20% at intervals of 2% (Figure 15). The graph shows that, even at high packet loss probabilities, the number of retransmitted packets was in the range of 11–13%.

**Figure 16:**
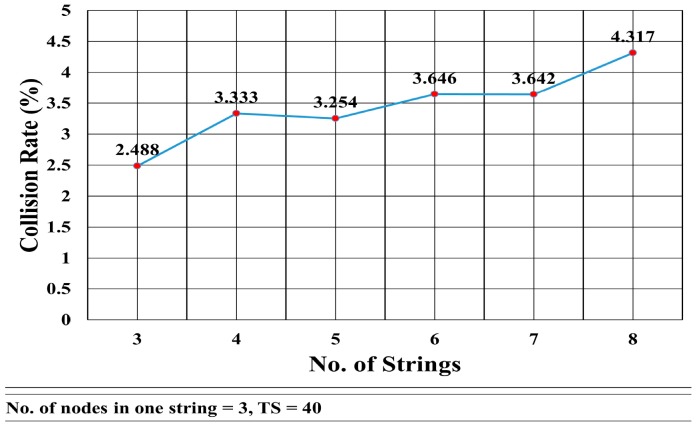
Percentage of Collision Rate vs. No. of Strings.

The collision rate is also tested. Figure 16 shows the collision rate when the number of nodes is constant (three nodes in each string), whereas the number of strings increases from three to eight. The graph shows that the collision rate increases nonlinearly with the number of strings. It shows that there is a slight change in the collision rate when the increase in the number of strings is from an odd number to an even number. From even number of strings to the odd number of strings, the change is negligible.

**Figure 17:**
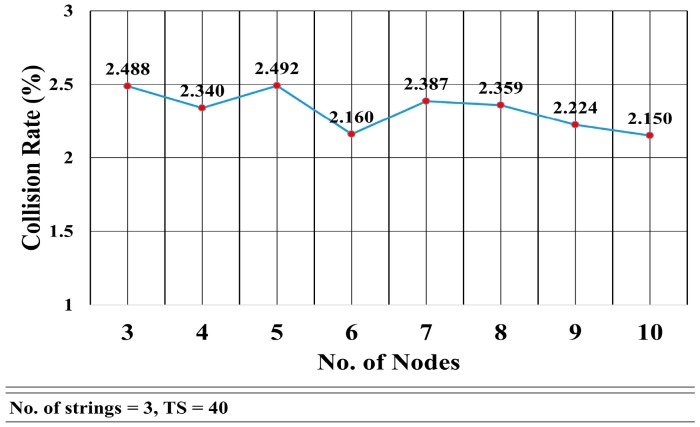
Collision Rate vs. Number of Nodes.

Figure 17 shows the collision rate as the number of nodes per string increases from 3 to 10. There is no clear relationship between the number of nodes in a string and the collision rate. It seems that the collision rate remains roughly constant. The mean is μ = 2.325 and the standard deviation was σ = 0.135. This was due to the fact the collision among the nodes mainly depends on the number of strings rather than the number of nodes in a string.

**Figure 18:**
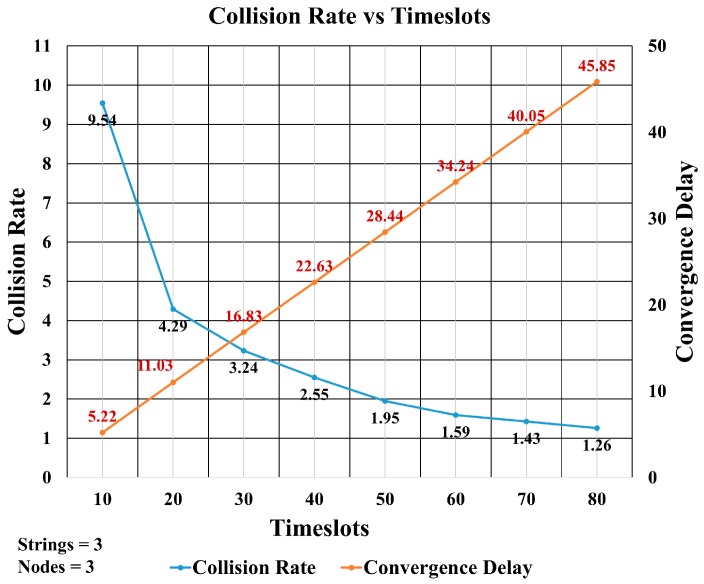
Collision Rate vs. Convergence Delay.

The number of timeslots is an important factor in controlling the collisions between transmitted packets. Figure 18 shows the simulation results for different numbers of timeslots ranging from 10 to 80. This comparison shows the tradeoff between collision rate and convergence delay. It is clear from the graph that increasing the number of timeslots decreased the collision rate, but it increased the convergence delay. When using 10 timeslots, the convergence delay is minimum but the collision rate is maximum, whereas in case of 80 timeslots, the number of collisions was at a minimum but the convergence delay was at a maximum. From Figure 18, it is observed that the optimum number of timeslots is around 30. We have chosen 40 timeslots in order to have a low collision rate, though at the cost of a little higher convergence delay.

**Figure 19:**
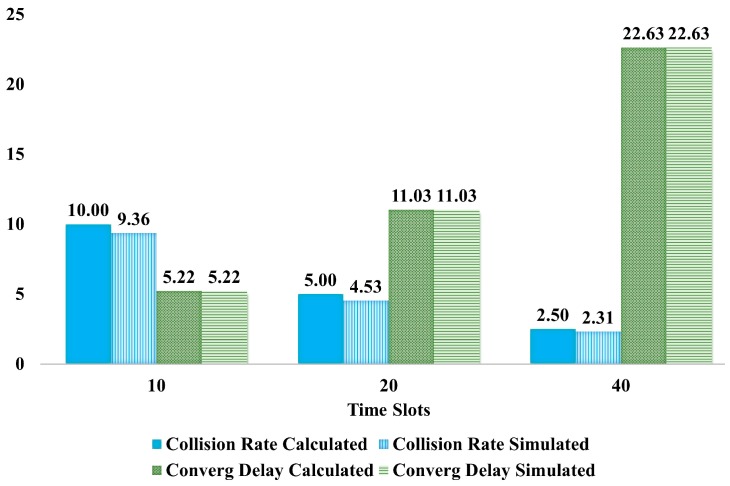
Comparison of Time Slots for Collision Rate and Convergence Delay.

Figure 19 compares the convergence delay obtained from the mathematical equations and from the simulations (see green bars). It can be seen that there was almost perfect agreement between these values. Figure 19 also includes a comparison between the simulated collision rate values and the calculated collision rate. It also shows that the simulated collision rate was in agreement with the theoretical analysis.

**Figure 20:**
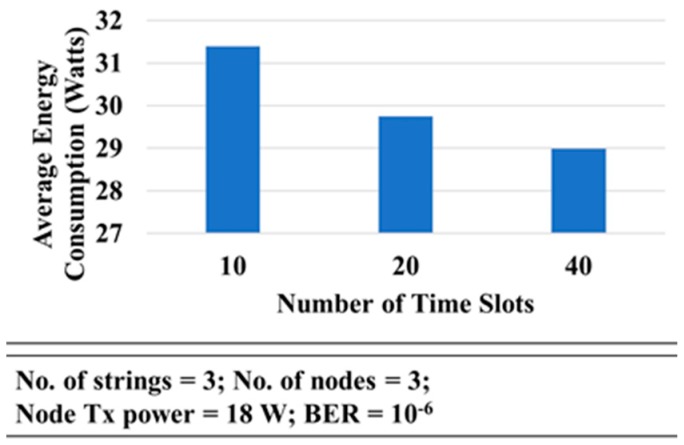
Energy Consumption vs. Number of Time Slots.

It can be seen in Figure 20 that the average energy consumption for 10 timeslots was higher than for 20 or 40 timeslots. This happens because the number of collisions in case of 40 timeslots was lower than in the other scenarios and, as a result, the number of retransmitted packets was lower.

### Discussion of Results

If we analyze the results, we can see that the convergence delay was due to the long propagation delay of the underwater acoustic channel. In order to avoid collisions, some kind of time synchronization must be used in this protocol. For example, it is possible that RR packets sent by the first nodes of the side strings have been successfully received by the second nodes but we have to wait for a period equal to three transmissions of RR and RR_ACK packets before the side nodes transmit the CF_RR packet to the intermediate nodes. The reason is that the two side nodes do not know about each other transmission’s state. If one side node has successfully sent the RR packet but the other one needs one more transmission, then synchronization will be lost, which may cause collisions at later stages. 

Figure 14 shows that the convergence delay increases linearly with the number of nodes in the strings. In Figure 13, we see that the convergence delay changed only when the number of strings increased from even to odd. Figure 16 shows that the collision rate also increased substantially when the number of strings grew from odd to even, whereas there is a trivial increase in the collision rate when the number of strings grows from even to odd. However, Figure 16 shows that, when the number of nodes increased, the collision rate remained almost the same with an average of 2.325% and standard deviation of σ = 0.135. 

The percentage of retransmitted packets increased with the probability of packet loss but it was not high (see Figure 15). Under normal channel conditions, we may expect low packet loss probability and hence a small number of packet retransmissions.

We can compare our proposal with the location-free protocols described in Section 2.1. Both NLPU [16] and SOFRP are proactive protocols, using a probe message to establish the path and their topology has one gateway node. However, there are two main differences: (a) NLPU does not replicate the physical topology, thus paths may be longer than required; and (b) the channel is split into several sub-channels, thus decreasing the available bandwidth per node.

Assume a network that has 30 nodes, organized in 5 strings and 6 rows. The distance between the nodes is 1000 m and data rate is 10 kbps. The communication channel is divided into seven subchannels, each with a data rate of 1428 bps. If we analyze the end-to-end delay achieved with NLPU and SOFRP when the farthest node transmits, we find that for NLPU, this value is 20.3 s, while SOFRP achieves an end-to-end delay of 4.3 s. Overall, an improvement of roughly 75%.

It is not possible to carry out a proper comparison with the other location free protocol, LFLSR, as the results shown in Reference [17] do not describe the network configuration and information on the number of hops each packet requires is missing. However, the use of periodic Hello packets imposes an additional burden that increases the end-to-end delay of packets. Hence, LFLSR would at least require 0.667 s more than SOFRP per each Hello packet transmission that the data packet encountered along the way.

## 8. Conclusions

In this paper, we have presented a routing protocol adapted for acoustic underwater networks with a hybrid radial/linear topology. It can also be easily used in hexagonal or rectangular grids by just allowing the formation of multiple connections via one intermediate node. Such networks are an interesting topology for monitoring medium-size oceanic regions such as estuaries, fishing zones, or geological features of interest. The protocol is designed to minimize the delay for sensitive data transmitted by the nodes, minimize the costs of managing such a network, and to provide strong resilience. We have reduced the energy consumption by designing the protocol in such a way that we avoid collisions that may occur during the initialization and maintenance of routes. Furthermore, it does not require additional sensing devices. 

As a proactive protocol, this proposal achieves a low packet forwarding latency compared to reactive protocols. Although the convergence time at initialization is high due to the effort made in the design to avoid collisions, once the routing paths have been formed, the packet forwarding delay is quite low, thus reducing computational cost. 

The gateway initiates the path formation process, assigns a unique identification for each string, and serves as a connecting point with external networks. The paths are created in a hybrid radial/linear way, by forwarding a Route Request packet that travels along the network and, upon reaching the end of each string, a response is sent back. Cross-layer techniques have been used to minimize the odds that some node is mistakenly considered down during path initialization.

To counteract node failures, the protocol includes mechanisms for creating alternative paths, thus making the protocol fault-tolerant. The full set of possible scenarios has been analyzed in the paper. The protocol provides viable solutions for all of them without compromising the transmissions of the other nodes.

A performance analysis has also been presented, and it has been validated through simulations. The analysis of results shows that extending the network, both in number of nodes per string or in number of strings, has a linear impact on the performance.

## Figures and Tables

**Table 1:** Packet Descriptions.

Packet Name	Description	Code
BEACON	Beacon	1100011
CF_RR	Clear to Forward the RR	0101000
CFRR_ACK	CF_RR ACKnowledgement	0100001
CFRR_BACK	CFRR from intermediate node to the side nodes	1011100
CFRRBACK_ACK	CFRR_BACK ACKnowledgement	1000111
CNCL	CaNCeL	0010001
CNCL_ACK	CaNCeL ACKnowlegement	0010000
ENQ	ENQuiry	0111100
ENQ_ACK	ENQuiry ACKnowledgement	0111100
INFO_REQ	INFOrmation REQuest	0111010
INFOREQ_ACK	INFO_REQ ACKnowledgement	0111010
RR	Route Request	0000111
RR_ACK	Route Request ACKnowledgement	0100101
RR_RSP	RR ReSPonse	0101101
RRRSP_ACK	RR_ReSPonse ACKnowledgement	0001101
SGN	Search Gateway Neighbor	0101010
SGN_RSP	SGN ReSPonse	0011001
SGNRSP_ACK	SGN_ReSPonse ACKnowledgemnt	0001100
SHN	Search Horizontal Neighbor	0001011
SHN_ACK	SHN ACKnowledgment	0100000
ST_RSP	STart sending the RR ReSPonse	0110001
STRSP_ACK	ST_RSP ACKnowledegement	0110101

**Table 2:** List of the symbols used in the equations.

Symbol	Description
DCFRR	Delay for CF_RR and CFRR_ACK packets delivery
DCFRRBACK	Delay for CFRR_BACK and CFRRBACK_ACK packets delivery
DGWRR	Delay for RR packet delivery to the gateway
DRR	Delay for RR packet delivery start at first layer
D_RRALL_	Total delay for RR packet to be delivered to all nodes
DRRRSP	Delay for RR_RSP and RRRSP_ACK packets delivery
DSGN	Delay for SGN packet delivery
DSHN	Delay for SHN and SHN_ACK packets delivery
DSHNRSP	Delay for SHNRSP and SHNRSP_ACK packets delivery
D_TOTAL_	Total delay for RR_RSP packet to reach the gateway
DR	Data rate (bits/sec.)
DT	Distance between two nodes (m)
GT	Guard time (sec.)
LTS	Duration of a wait period (sec.)
ND	Number of nodes in a string
PKD	Packet transmission delay (sec.)
PROD	Propagation delay (sec.)
PS	Packet size (bits)
RPH	Repetition of RR packet forwarding within a layer
RPV	Repetition of RR packet forwarding through all layers
SPS	Sound Propagation Speed (m/s)
ST	Number of strings
TS	Duration of a timeslot (sec.)
TTS	Number of timeslots in a wait period
WP	Wait Period

**Table 3:** Simulation Parameters.

Parameter	Value	Unit
Sound speed	1500	m/s
Distance between nodes	500	m
Data rate	5000	bits/s
Header size	37	bits
Transmission power	18	Watts
Bit error rate	10^−6^	
Number of runs	500	

**Table 4:** Packet Delay Analysis.

Nodes (ND)	Packet Delay (PKD)	PKD_i_ − PKD_i-1_
3	0.0122	
4	0.0138	0.0016
5	0.0154	0.0016
6	0.0170	0.0016
7	0.0186	0.0016

**Table 5:** Convergence Delay Analysis.

Nodes (ND)	Convergence Delay (CD)	α = CD_i_ − CD_i-1_	β = α_i_ − α_i-1_
3	22.639344		
4	32.694753	10.055409	
5	42.841890	10.147137	0.091728
6	53.080755	10.238865	0.091728
7	63.411348	10.330593	0.091728
